# Supplementary material for: The causal role of circulating inflammatory markers in osteoporosis: a bidirectional Mendelian randomized study
Source: Front Immunol. 2024 Jul 18;15:1412298. doi: 10.3389/fimmu.2024.1412298 (PMC11291241; doi:10.3389/fimmu.2024.1412298)
Supplement: Supplementary file 1 [file DataSheet_1.zip › supplementary material/CIM-OSTEOPOROSIS /GCST90274787.csv_leave_one_out.pdf]

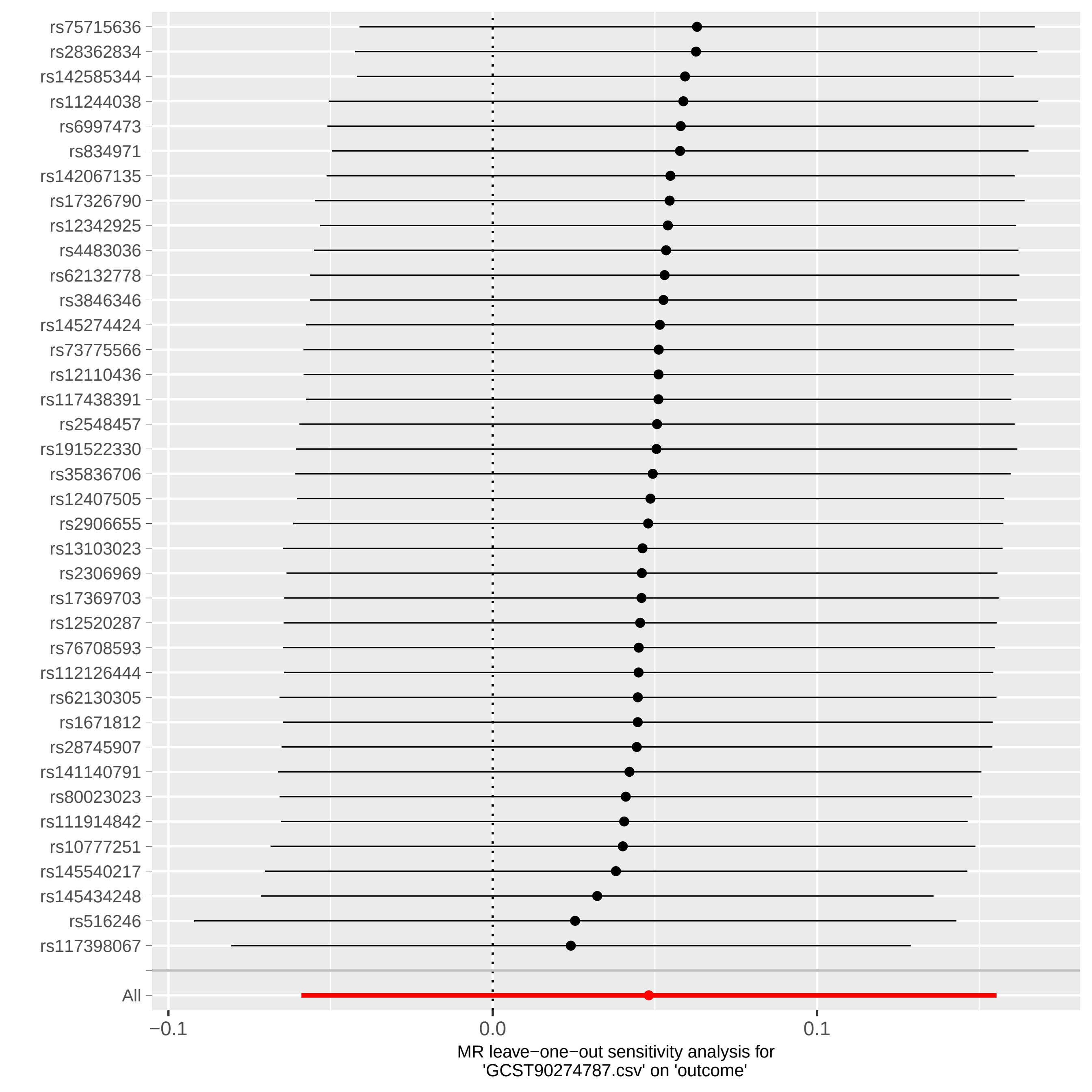
rs75715636
rs28362834
rs142585344
rs11244038
rs6997473
rs834971
rs142067135
rs17326790
rs12342925
rs4483036
rs62132778
rs3846346
rs145274424
rs73775566
rs12110436
rs117438391
rs2548457
rs191522330
rs35836706
rs12407505
rs2906655
rs13103023
rs2306969
rs17369703
rs12520287
rs76708593
rs112126444
rs62130305
rs1671812
rs28745907
rs141140791
rs80023023
rs111914842
rs10777251
rs145540217
rs145434248
rs516246
rs117398067
All
−0.1
0.0
0.1
MR leave−one−out sensitivity analysis for
'GCST90274787.csv' on 'outcome'
